# REC-2006—A Fractionated Extract of *Podophyllum hexandrum* Protects Cellular DNA from Radiation-Induced Damage by Reducing the Initial Damage and Enhancing Its Repair *In Vivo*

**DOI:** 10.1093/ecam/nep212

**Published:** 2011-06-16

**Authors:** Pankaj Chaudhary, Sandeep Kumar Shukla, Rakesh Kumar Sharma

**Affiliations:** ^1^Institute of Nuclear Medicine and Allied Sciences (INMAS), Delhi, India; ^2^Brookhaven National Laboratory, Upton, NY 11973, USA

## Abstract

*Podophyllum hexandrum*, a perennial herb commonly known as the Himalayan May Apple, is well known in Indian and Chinese traditional systems of medicine. *P. hexandrum* has been widely used for the treatment of venereal warts, skin infections, bacterial and viral infections, and different cancers of the brain, lung and bladder. This study aimed at elucidating the effect of REC-2006, a bioactive fractionated extract from the rhizome of *P. hexandrum*, on the kinetics of induction and repair of radiation-induced DNA damage in murine thymocytes *in vivo*. We evaluated its effect on non-specific radiation-induced DNA damage by the alkaline halo assay in terms of relative nuclear spreading factor (RNSF) and gene-specific radiation-induced DNA damage via semi-quantitative polymerase chain reaction. Whole body exposure of animals with gamma rays (10 Gy) caused a significant amount of DNA damage in thymocytes (RNSF values 17.7 ± 0.47, 12.96 ± 1.64 and 3.3 ± 0.014) and a reduction in the amplification of **β**-globin gene to 0, 28 and 43% at 0, 15 and 60 min, respectively. Administrating REC-2006 at a radioprotective concentration (15 mg kg^−1^ body weight) 1 h before irradiation resulted in time-dependent reduction of DNA damage evident as a decrease in RNSF values 6.156 ± 0.576, 1.647 ± 0.534 and 0.496 ± 0.012, and an increase in **β**-globin gene amplification 36, 95 and 99%, at 0, 15 and 60 min, respectively. REC-2006 scavenged radiation-induced hydroxyl radicals in a dose-dependent manner stabilized DPPH free radicals and also inhibited superoxide anions. Various polyphenols and flavonoides present in REC-2006 might contribute to scavenging of radiation-induced free radicals, thereby preventing DNA damage and stimulating its repair.

## 1. Introduction

Radiation-induced free radicals oxidize cellular biomacromolecules like DNA, proteins and lipids generating a variety of cellular dysfunctions leading to cell death [1, 2]. Damages to DNA, such as single- and double-strand breaks, base modifications and adduct formation, are considered as biologically significant cellular lesions [3–5]. Normally, cells operate diverse pathways to repair oxidative damage to DNA. Among them, the most important are direct repair of an adduct, base-excision repair, nucleotide-excision repair, homologous recombination, non-homologous end-joining, DNA inter-strand cross-link repair and DNA mismatch repair [6].

Several radioprotective agents, including amifostine, aminothiols, cysteamine, polyamines and DNA-binding ligands like Hoechst, protect DNA from radiation-induced damage [7, 8]. However, therapeutic levels of most of these agents entail severe side effects, such as nausea, vomiting, hypotension and neurotoxicity, thereby limiting their clinical use [9]. Consequently, newer and more effective agents are being sought. Recent reports suggest that various plant extracts and natural products protect DNA from radiation-induced oxidative damage [10–17].

*Podophyllum hexandrum* (also known as Himalayan May Apple), a herb thriving at high altitudes in the Himalayas has been extensively used in Ayurvedic system of medicine for the treatment of ailments like monocytoid leukemia, Hodgkin's lymphoma, bacterial and viral infections, venereal warts, rheumatoid artharalgia associated with limb numbness and pycnogenic infections of skin tissue [18–20]. The root and rhizome of *P. hexandrum* are reported to contain a number of compounds with significant pharmacological properties, for example, epipodophyllotoxin, podophyllotoxone, 4-methylpodophyllotoxin, aryltetrahydronaphthalene lignans, flavonoids such as quercetin, quercetin-3-glycoside, 4-demethylpodophyllotoxin glycoside, podophyllotoxinglycoside, kaempferol and kaempferol-3-glucoside [21, 22].

An extract (code named as RP-1) from the rhizome of *P. hexandrum* reportedly protects mice against a lethal dose of ionizing radiation [23]. Different mechanisms were proposed to account for these radioprotective properties, including free radical scavenging, metal chelation and the elevation of antioxidant defense enzymes [24, 25]. A variety of scenarios involve radiation exposures in the moderate range (i.e., 1–10 Gy), including cancer therapy, planned reactor maintenance and the explosion of a dirty bomb (radioactive dispersal device). With the likelihood that exposure to a moderate radiation dose will result in radiation-induced DNA damage entailing cell death, we undertook this study to assess the effect of REC-2006 [25], a fractionated extract of rhizome of *P. hexandrum,* on the induction and repair kinetics of radiation (10 Gy)-induced gene-specific and non-specific DNA damage in murine thymocytes *in vivo*.

Intraperitoneal administration of REC-2006 to mice at 15 mg kg^−1^ body weight conferred more than 90% protection against whole-body irradiation (10 Gy) as compared to 72% offered by 34.5 mg kg^−1^ body weight administration of the parent plant extract RP-1. The radiosensitive nature of thymocytes provides an attractive system to study DNA repair [26, 27]. As we are interested to look at the early kinetics of DNA repair within 1 h after irradiation, we choose mouse *β*-globin gene which is constitutively expressed in transcriptionally active or inactive domains thus acting as a biomarker of overall DNA repair [28, 29]. Similar approach has also been used for investigating DNA damage repair [13, 30].

Observations of the effect of *P. hexandrum* upon the repair kinetics of gamma-radiation-induced DNA damage will further delineate the mechanisms involved in overall radioprotective effects of this plant.

## 2. Methods

### 2.1. Chemicals

We obtained the following materials from Sigma Chemical Co. (St. Louis, MO, USA): agarose, low melting point agarose, aluminium trichloride, bromophenol blue, dimethyl sulfoxide (DMSO), di-sodium ethylene di-amine tetra acetic acid (Na_2_-EDTA), 2,2-diphenyl-1-picrylhydrazyl (DPPH), ethidium bromide, gallic acid, quercetin, sodium chloride (NaCl), sodium hydroxide (NaOH), sodium lauryl sarcosine, sucrose, triton-X-100, Tris—HCl, Tris base, trichloroacetic acid (TCA), thiobarbituric acid (TBA) and xylene cyanol. Taq polymerase and dNTP mix were purchased from Qiagen, Chatsworth, CA. Primers were synthesized from The Center for Genomic Application, Delhi, India. All other chemicals and reagents used in this study were of high purity.

### 2.2. Collection and Processing of Plant Material

REC-2006, the chloroform-extracted fraction of *P. hexandrum*, was prepared following the method of Gupta et al. [25]. Briefly, powdered material from the rhizome of *P. hexandrum* was transferred to a Soxhlet apparatus and consecutively extracted with chloroform for a minimum of three times over 24–72 h using a proportionate amount of solvent. The pooled filtrates were filtered through Whatman paper no. 3, concentrated by solvent evaporation under reduced pressure in a rotary evaporator (Buchi, Switzerland) and dried. The dried powder so obtained was code-named REC-2006. The crude extract (RP1) was made at INMAS whereas help of Regional Research Laboratory, Jammu, India was obtained in further fractionations and photochemical analysis. Code name has been assigned to the extract to distinguish it from other fractions of the parent extract and to prevent any biasness in studies.

### 2.3. Animals

Swiss albino strain “A” male mice (10–12 weeks) weighing 25 ± 2 g were maintained under standard laboratory conditions (25 ± 2°C; photoperiod 12 h light/dark cycle) and fed standard animal food pellets (Amrut Laboratory Feed, Delhi, India) with water *ad libitum*. Permission for animal experimentation was obtained from the Institutional Animal Ethics Committee, and all the guidelines pertaining to use and care of animals were followed strictly, as required by the Central Drug Research Institute, Lucknow, India.

### 2.4. Irradiation

Animals from each group, kept in a perforated acrylic box, were irradiated according to experimental requirements using ^60^Co Gamma cell model-220 (Atomic Energy Commission, Ontario, Canada) with a dose rate of 0.312 Gy min^−1^. 

### 2.5. Drug Administration and Experimental Plan

REC-2006, tested negative for endotoxins, dissolved in 8% DMSO and a radio protective concentration of 15 mg kg^−1^ body weight was injected intraperitoneally to the different groups, 1 h before exposing the mice to 10 Gy gamma rays (the lethal dose) following the experimental plan shown in Table 1.

### 2.6. Isolation of Thymocytes

Mice were killed by cervical dislocation, dissected, the abdominal cavity was perfused with 0.9% saline and the thymus was removed. All visible clots were segregated carefully. We minced the thymic lobes finely, and gently crushed them with the plunger of a syringe; the resultant cell suspension was passed through a 25-gauge needle to avoid cell aggregates. All procedures were carried out on ice.

### 2.7. Alkaline Halo Assay

We detected DNA single-strand breaks in individual cells using the alkaline halo assay [31] modified for murine thymocytes. After the different treatments, thymocytes were suspended (1.5 × 10^4^ to 2.0 × 10^4^ per 100 *μ*l) in a 1.5% low-melting agarose solution in phosphate buffered saline, pH 7.4, and immediately pipetted on to slides coated with 1.0% normal agarose and spread uniformly. After gelling was complete, the slides were immersed in the alkali buffer (0.1 M NaOH and 1 mM EDTA; pH 13) for 20 min at 4°C, washed, and then stained with 10 *μ*g ml^−1^ ethidium bromide for 5 min. The images were acquired on a fluorescent microscope (Leica Q550 FW, Wetzlar, Germany) equipped with a 40× Neofluar objective lens, and analyzed using Comet score software (Tritek Corporation, Northern Virginia, USA). The intensely stained, intact chromatin mass forms the central core of the halo, while the broken DNA fragments constitute its diffusely stained periphery. Levels of DNA damage were expressed as the relative nuclear spreading factor (RNSF) values, calculated by subtracting the RNSF values of control cells from those of treated cells [13]. 

### 2.8. DNA Isolation from Thymocytes

The genomic DNA from murine thymocytes (5 × 10^6^) was isolated using the DNeasy isolation kit (DNeasy Tissue kit, Qiagen, Chatsworth, USA) as per manufacturer's instructions. DNA yields were estimated spectrophotometrically by measuring absorbance at 260 and 280 nm.

### 2.9. Semi-Quantitative Polymerase Chain Reaction

Primer pairs used against mice *β*-globin 957 (5′-CGGGTGAGAGATACATCCATCG-3′) and *β*-globin 5638 (5′-GATCCAGAGAGCAACTTTCGACTA-3′) targeted the genomic *β*-globin gene cluster and yielded a product of 4681 bp [32]. The reaction mixture (20 *μ*l) contained template DNA (20 ng), dNTP mix (200 *μ*M each dNTP), 10 pM *μ*l^−1^ primer pairs, 0.3 U Taq polymerase, Taq buffer containing MgCl_2_ (1.5 mM) and sterile H_2_O. The polymerase chain reaction (PCR) cycling conditions used to amplify *β*-globin were pre-PCR incubation at 94°C for 2 min followed by 30 cycles of 94°C for 15 s, 59°C for 30 s and 68°C for 4 min. At the end of the profile, we added a final extension of 4 min at 72°C; the samples were stored at 4°C. An aliquot of each PCR product was resolved by agarose-gel electrophoresis [33]. Briefly, 10 *μ*l of the sample was mixed with 5 *μ*l of loading dye (0.025% bromophenol blue, 0.25% xylene cyanol and 40% sucrose in water) and loaded into the wells of a 1.2% agarose gel along with the 1 kb DNA ladder (Fermentas, MD, USA) to confirm the size of the PCR-amplified product. Electrophoresis was carried out for 2.5 h at 5 V cm^−1^ in the Tris acetate buffer in the presence of 0.5 *μ*g ml^−1^ ethidium bromide. The amplified products were quantified densitometrically using Image Quant software (Molecular Dynamics, Sunnyvale, CA).

### 2.10. DPPH Radical Scavenging Activity

We measured the 2,2-diphenyl-1-picrylhydrazyl (DPPH) radical-scavenging ability of REC-2006 according to the method of Shimada et al. [34]. A solution of DPPH in methanol mixed with REC-2006 was incubated for 15 min in the dark at 37°C. The decrease in absorbance at 517 nm was measured against that of methanol alone. The absorbance of DPPH alone was taken as 100% radical or 0% inhibition.

### 2.11. Hydroxyl Radical Scavenging

We quantified scavenging of radiation (100 Gy) and Fenton reaction induced hydroxyl radicals by REC-2006, using 2-deoxyribose as the marker substrate [35]. Briefly, we exposed to 100 *μ*M FeSO_4_ or 100 Gy, 1 ml of reaction volume containing 5 mM 2-deoxyribose in the absence or presence of varied concentrations of REC-2006. Thereafter, we added two volumes of solution containing 25% TCA and 1% TBA in 0.1 N NaOH, placed the mixture in a boiling water bath for 20 min, cooled it and measured the absorbance of the resulting pink-colored chromogen at 532 nm.

### 2.12. Estimation of the Scavenging of Chemically Generated Superoxide Anions

Following the method of Rao et al., we estimated the superoxide anion scavenging ability of REC-2006 [36]. Briefly, to a reaction mixture containing 0.52 M sodium pyrophosphate (pH 8.3), 186 *μ*M phenyl methane sulfate (PMS) and 300 *μ*M nitro blue tetrazolium (NBT), we added various concentrations of REC-2006. The reaction was initiated by adding nicotinamide adenine dinucleotide reduced (NADH) (final concentration 780 *μ*M) and incubating the solution for 90 s at 30°C. The purple-colored chromogen that formed was measured spectrophotometrically at 560 nm.

### 2.13. Total Antioxidant Capacity

The total antioxidant capacity of REC-2006 was determined spectrophotometrically by quantifying the amount of phosphomolybdenum complex generated [37]. A 0.1 ml (100 *μ*g) sample of REC-2006 was mixed with reagent solution (6 M sulfuric acid, 28 mM sodium phosphate and 4 mM ammonium molybdate), and the mixture was incubated at 95°C for 90 min. Thereafter, the samples were cooled to room temperature and the absorbance was measured at 695 nm against a blank solution (typically, incubating 1 ml of reagent and the appropriate volume of the same solvent (DMSO) used to prepare the samples under the same conditions as the experimental samples). The total antioxidant capacity of REC-2006 was expressed as gallic acid equivalents using a standard curve prepared from a freshly prepared gallic acid solution.

### 2.14. Estimation of the Total Phenolic Content

A 100 *μ*l aliquot of 10 mg ml^−1^ REC-2006 was mixed with 500 *μ*l Folin-Cioccalteau reagent and 400 *μ*l 7.5% sodium carbonate [38]. Following incubation at 20°C for 30 min, the absorbance was read at 765 nm. The total phenols in REC-2006 were expressed as gallic acid equivalents, estimated from a standard curve obtained from the absorbance of fresh gallic acid equivalents. 

### 2.15. Total Flavonoid Content

We obtained the total flavonoid content of REC-2006 using the method of Zhisen et al. [39]. To 1 ml of diluted REC-2006 sample (500 *μ*g), we added 4 ml of H_2_O, 0.3 ml of 5% NaNO_2_ followed, 5 min later, by of 0.3 ml of 10% AlCl_3_ solution. One minute thereafter, 2 ml 1 M NaOH was added and the reaction mixture was immediately diluted with 2.4 ml of ddH_2_O. The pink-colored chromogen was measured spectrophotometrically at 510 nm. The total flavonoid content (mg mg^−1^) was expressed as quercetin equivalents, using a standard curve from a fresh quercetin solution.

### 2.16. Data Analysis

The data are presented as the mean ± standard deviation (SD) of three separate experiments, with each experiment comprising three parallel measurements. We compared radiation and radiation + REC-2006 groups. The data were analyzed by one-way analysis of variance, and multiple comparisons were made between different groups by applying Bonferroni *t*-test. A probability of < 5% was considered significant.

## 3. Results

### 3.1. Phytochemical Analysis

**Figure 1:**
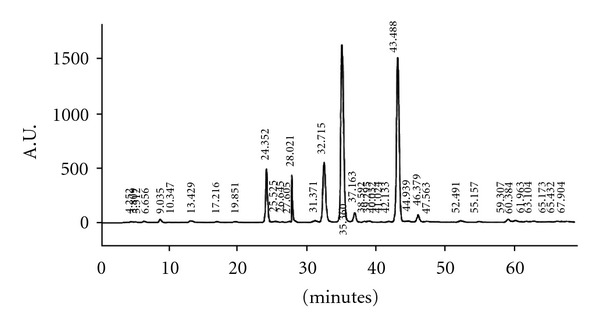
HPLC profile of REC-2006.

Figure 1 shows the HPLC profile of REC-2006. Demethylpodophyllotoxin, podophyllotoxin glycoside, epipodophyllotoxin and podophyllotoxin among others were identified by analyzing the fragmentation patterns [25]. Chemical analysis and spectrophotometric determinations indicated that the total polyphenol content of REC-2006 was 8 mg mg^−1^ of gallic acid equivalents while the total flavonoid content was 0.20 mg mg^−1^ of quercetin equivalents.

### 3.2. REC-2006 and Radiation-Induced DNA Damage

#### 3.2.1. Non-Specific DNA Damage and Repair

**Figure 2:**
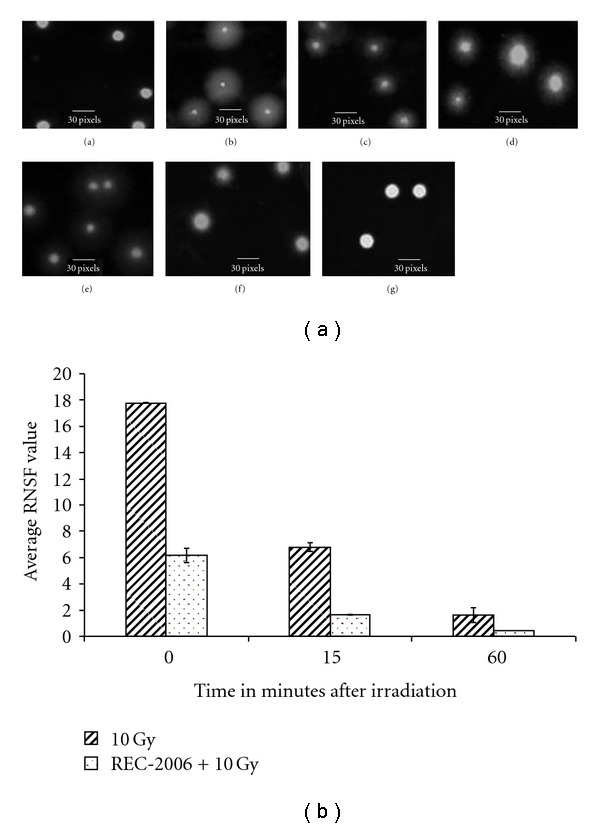
(a) Effect of REC-2006 on 10 Gy-induced single strand breaks in mouse thymocytes. Mice were irradiated with or without REC-2006 treatment (8 mg kg^−1^ body weight i.p. 60 minutes before irradiation) and thymocytes from them were isolated at different intervals. DNA damage was studied employing the alkaline halo assay. (a) Cells without any treatment. (b–d) Cells from 10 Gy irradiated mice killed after 0, 15 or 60 min, respectively. (e–g) Cells from REC-2006 pretreated irradiated mice killed after 0, 15 or 60 min, respectively. (b) Quantitative estimation of REC-2006 effect on radiation-induced DNA damage and repair. The level of DNA strand breakage was quantified by calculating the nuclear spreading factor value, which is the ratio of the area occupied by the halo (obtained by subtracting the area of the nucleus from the total area of the nucleus + halo) to the area occupied by the nucleus. Data are expressed as the relative nuclear spreading factor (RNSF), which was calculated by subtracting the nuclear spreading factor values of control cells from those of treated cells. Data are the mean ± SD for at least 100 cells for each observation in triplicate.

Effect of REC-2006 on DNA single strand breaks is evaluated through alkaline halo assay formation as shown in Figure 2(a) (a–g). Control cells showed intact nuclei without any halo around them (a), while 10 Gy whole body irradiation, resulted in large halo formation (b). The center of the halo became a little intense and bigger in radius at 15 and 60 min after irradiation (c and d). Thymocytes from REC-2006 pre-treated whole body irradiated mice revealed more intense and bigger nuclei from 0 min time point onward and at 15 and 60 min after irradiation the halo could not be seen, only intact nucleus was seen. The level of DNA strand breakage was quantified by calculating the nuclear spreading factor value, which is the ratio of the area occupied by the halo (obtained by subtracting the area of the nucleus from the total area of the nucleus + halo) to the area occupied by the nucleus. Data are expressed as the RNSF, which was calculated by subtracting the nuclear spreading factor values of control cells from those of treated cells. Untreated cells consistently had a nuclear spreading factor of zero. In un-irradiated mice, the RNSF, an indicator of DNA damage, was zero. In the group receiving radiation alone, the average RNSF immediately after irradiation was 17.7 ± 0.47. However, with increasing time, the amount of DNA damage decreased; RNSF values after 15 and 60 min, respectively, were 12.96 ± 1.64 and 3.3 ± 0.014 (Figure 2(b)). In animals pretreated with REC-2006 and then exposed to 10 Gy, the amount of initial radiation-induced DNA damage was significantly lower (6.156 ± 0.576) than in the radiation-alone group; the average RNSF values at 15 and 60 min afterward were, respectively, 1.647 ± 0.534 and 0.496 ± 0.012 (Figure 2(b)).

#### 3.2.2. Gene-Specific DNA Damage and Repair

**Figure 3:**
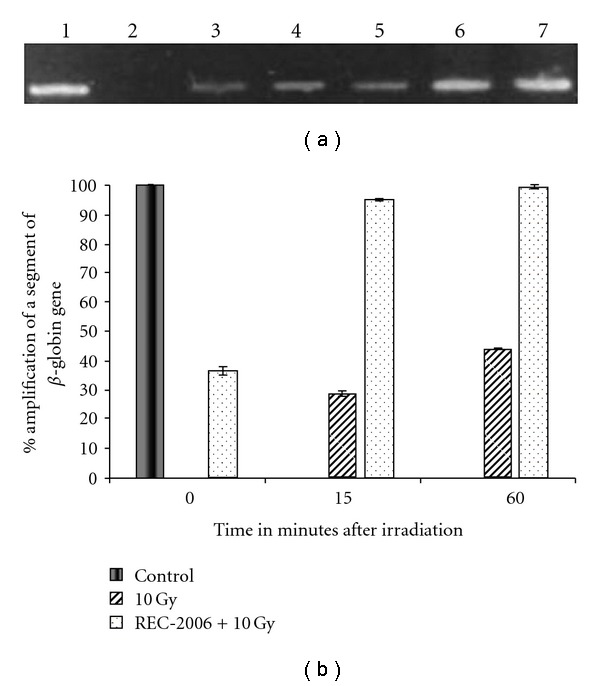
(a) Effect of REC-2006 on radiation DNA damage and repair in mouse thymocytes. Lane 1, PCR products from mice without any treatment; lanes 2–4, amplification product from mice exposed to 10 Gy and killed at 0, 15 and 60 min, respectively; lanes 5–7 amplification product from REC-2006 pretreated irradiated mice taken at 0, 15 and 60 min, respectively. (b) Quantitative estimation of effect of REC-2006 on repair kinetics of *β*-globin gene in mice *in vivo*. Graph shows the percent amplification of *β*-globin in the absence or presence of REC-2006 at 0, 15 and 60 min after 10 Gy irradiation to mice.

We first carried out studies with varied concentrations of DNA from untreated control cells to find the appropriate amount of template DNA to use; we found a linear relationship between the template concentration and sequence amplification up to 30 ng DNA. In our further studies of the effect of REC-2006 on radiation-induced gene-specific DNA damage and repair, we selected a template concentration of 20 ng. After amplification, the size of PCR product was determined by running the PCR product with a broad range (1 kb) DNA marker (figure not shown). Semi-quantitative polymerase chain reactions for the irradiation-alone group (Figure 3(a), lane 2) revealed a complete loss of amplification of the *β*-globin gene immediately after exposure (0 min), indicating severe damage to the template DNA. After 15 or 60 min, the quantum of DNA damage decreased and the amplification levels were, respectively, 28 and 43% of the unirradiated controls (Figure 3(a), lanes 3 and 4, and Figure 3(b)). However, REC-2006 pretreatment significantly lowered the radiation-induced sequence-specific DNA damage at all post-irradiation intervals in comparison to the irradiation-alone group (Figure 3(b)). The amplification products of the *β*-globin gene sequence in the REC-2006 pretreated irradiated group was 36, 95 and 99%, respectively, at 0, 15 and 60 min after 10 Gy exposure (Figures 3(a), 3(b), lanes 5, 6 and 7).

### 3.3. Hydroxyl- and DPPH-Radical Scavenging by REC-2006

**Figure 4:**
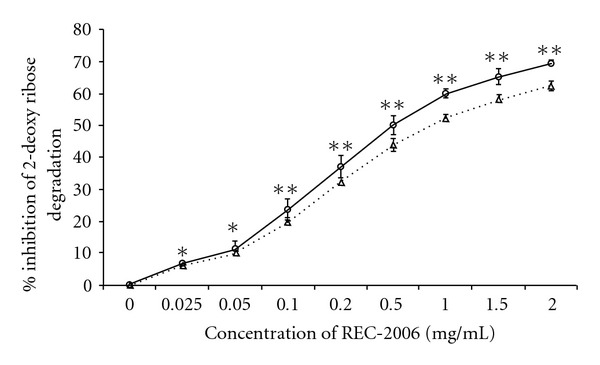
Effect of REC-2006 on radiation-induced hydroxyl generation and subsequent degradation of 2-deoxyribose, as measured by percentage inhibition in the formation of TBARS. Briefly, 1 ml of reaction volume containing, 5 mM 2-deoxyribose and varied concentrations of REC-2006 were mixed either with 100 *μ*M FeSO_4_(Fenton) or exposed to 100 Gy followed by incubation for 1 h at 37°C. There after, two volumes of solution containing 25% TCA and 1% TBA in 0.1 N NaOH was added, incubated in boiling water bath for 20 min, cooled and the absorbance of the resulting pink-colored chromogen was measured at 532 nm. The data in this figure represent the mean ± SD of values from three independent assays (*P < .05, **P < .01).

**Figure 5:**
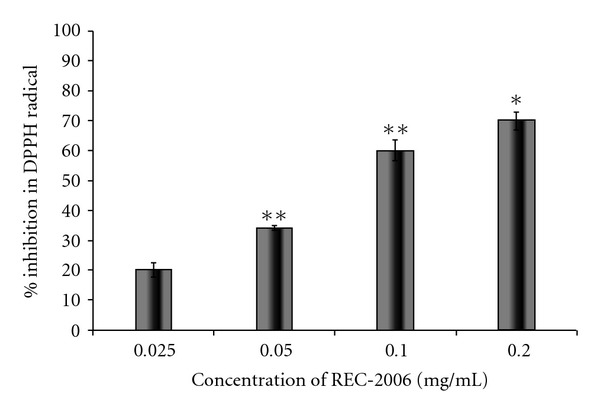
Effect of REC-2006 on DPPH radical scavenging. Methanolic solutions of DPPH and REC-2006 were mixed. Thereafter, the samples were incubated for 15 min in dark at 37°C and the decrease in the absorbance at 560 nm was measured against methanol. The absorbance of DPPH alone was considered as 100% radical or 0% inhibition. The values are expressed as mean ± SD of data from three independent assays (*P < .05, **P < .01).

REC-2006 scavenged, in a dose-dependent manner, hydroxyl radicals generated either by the Fenton reaction or by radiation (100 Gy) (Figure 4). Up to 0.05 mg ml^−1^ REC-2006 gradually inhibited the 2-deoxy-ribose degradation and the difference in inhibition was not very significant in Fenton-mediated and 100-Gy-induced hydroxyl radicals. Beyond 0.05 mg ml^−1^, a significant difference was observed in inhibition. Maximum inhibition was observed at a concentration of 2.0 mg ml^−1^ for radiation (62.5%) and Fenton reaction (69.2%)-mediated 2-deoxyribose degradation. Increasing concentrations of REC-2006 significantly stabilized the DPPH radicals in a dose-dependent fashion maximally at 0.2 mg ml^−1^ (Figure 5). The absorbance of DPPH alone was taken as 100% radical or 0% inhibition. Beyond 0.2 mg ml^−1^, a plateau phase was observed (not shown in the figure).

### 3.4. Super Oxide Anions Scavenging Potential, Total Antioxidant Capacity, Polyphenol and Flavonoid Contents of REC-2006

**Figure 6:**
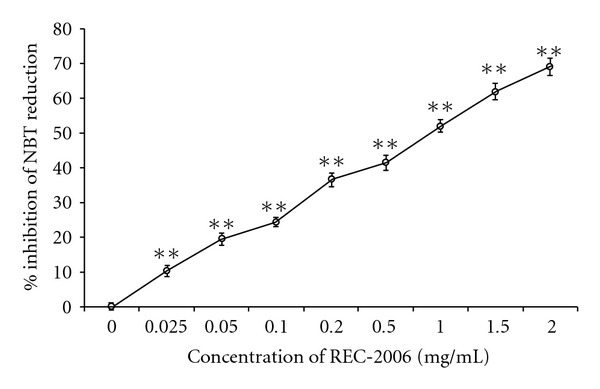
Effect of REC-2006 on chemically induced superoxide anions. The reaction mixture containing of 0.52 M sodium pyrophosphate, pH 8.3, 186 *μ*M phenazine methosulphate, 300 *μ*M nitroblue tetrazolium and 780 *μ*M NADH was mixed with varied concentrations of REC-2006 individually and incubated for 90 s at 30°C. The purple-colored chromogen formed was measured spectrophotometrically at 560 nm in triplicate. The values are expressed as mean ± SD of data from three independent assays (NS, not significant; *P < .05, **P < .01).

The superoxide anions generated by phenyl methane sulfate and NADH reduced NBT. REC-2006 inhibited the chemically generated superoxide anion formation in a concentration-dependent fashion. Maximum scavenging (absorbance 0.056) was observed at concentration of 1.5 mg ml^−1^ (Figure 6). The highest concentration of REC-2006 we assayed was 2 mg ml^−1^ and beyond that a plateau effect was observed. The total antioxidant capacity of the extract was 0.06 mg mg^−1^ gallic acid equivalents.

## 4. Discussion

The requirement of administering *P. hexandrum* before irradiation to achieve radioprotection suggests that mechanisms such as free radical scavenging, metal chelation and elevation of antioxidant defense systems might have a major role. However, a very large quantum of the drug/protective agent must be present in the cellular milieu to scavenge the heavy flux of free radicals generated by high doses of radiation and cannot be achieved under normal circumstances. One of the important aspects of post-irradiation cell recovery is the repair of radiation-induced oxidative DNA damage.

We explored the total genomic DNA damage and repair in mouse thymocytes using the alkaline halo assay, finding that treatment with REC-2006 significantly decreased radiation-mediated DNA strand breaks (Figures 2(a) and 2(b)). As the mice were killed immediately after radiation, and samples were processed at 4°C throughout, the role of repair in the initial decrease in DNA damage (0 min) can be ruled out. We consider that the decline in this DNA damage occasioned by REC-2006 treatment might be attributable to the free-radical scavenging or antioxidant potential of the flavonoides and polyphenolics present in the extract.

To gain insight into the mechanism of DNA protection, we evaluated *in vitro* the effect of REC-2006 on the scavenging of radiation-induced hydroxyl radicals, and DPPH radicals scavenging, on the generation of superoxide anions, and on the total antioxidant potential. The inhibition of 2-deoxyribose degradation by oxidants, a simple, reliable technique to assess the hydroxyl scavenging ability of an agent [12], clearly demonstrated the hydroxyl radical scavenging potential of REC-2006 (Figure 4). Its potential for scavenging of free radicals was further supported by its inhibition of DPPH and superoxide anions (Figures 5 and 6). Total antioxidant potential evaluated in gallic-acid equivalents attested to its antioxidant function. We assessed the amount of radiation-induced DNA damage remaining at various times after exposure to evaluate the effect of REC-2006 on cellular repair. In mice given REC-2006 before irradiation, this measure seemingly led to faster DNA repair; thus, after 15 and 60 min, their RNSF values were, respectively, 1.647 ± 0.534 and 0.496 ± 0.012 in comparison to a group not given REC-2006 (12.96 ± 1.64 at 15 min or 3.3 ± 0.014 after 60 min) (Figure 2(b)).

To better elucidate the radioprotective effect of REC-2006, DNA damage and repair was studied at gene level, choosing *β*-globin as a model gene and semi-QPCR. Previously, other investigators used *β*-globin or other constitutively expressed genes as marker genes for studying DNA damage and repair kinetics [40]. The rationale of the QPCR technique is that certain DNA lesions block the movement of the Taq polymerase on the DNA template, so decreasing its amplification [40, 41]. In the present study, we do not state any direct relationship between repair and protection in *β*-globin gene in thymocytes and amelioration of adverse effects of radiation; however, the only rationale of studying *β*-globin gene in thymocytes is based on the fact that radiosensitive nature of thymocytes makes them a highly sensitive system to study DNA repair [26, 27] and constitutive expression in transcriptionally active and inactive regions of *β*-globin makes it a suitable target gene [28, 29]. Exposing the mice to 10 Gy resulted in extensive DNA damage in the *β*-globin gene and complete immediate loss of amplification after exposure (Figures 3(a) and 3(b)). REC-2006 treatment significantly reduced the induced damage as evidenced by a 36% amplification compared to untreated controls. These results clearly indicate the radioprotective effect of REC-2006 *in vivo*. After 15 min, only 28% amplification was observed in the radiation-alone group whereas in the REC-2006-treated group most of the damage was repaired (95% amplification with respect to the control). Furthermore, after 60 min the amplification level in mice treated with REC-2006 was almost equal to that of controls (99%), pointing to an enhancement in the activity of the repair machinery. Meanwhile, there was only 43% amplification in the *β*-globin gene in the radiated group that had not received REC-2006.

**Figure 7:**
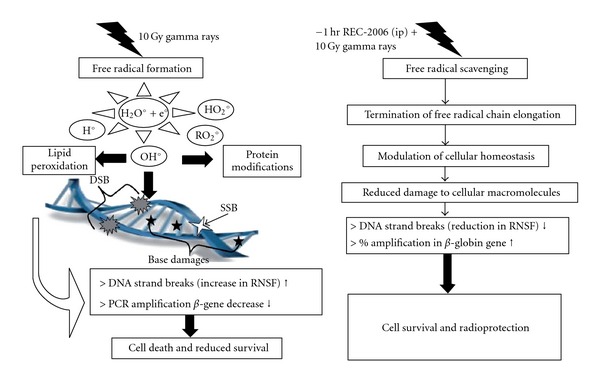
Proposed mechanism of action of REC-2006 against radiation-mediated DNA damage.

Both non-specific and gene-specific DNA damage repair studies clearly revealed that REC-2006 protects the cellular DNA from radiation-induced damage both by inhibiting the induction of damage and by enhancing its repair after exposure. The proposed mechanism of action of REC-2006 to protect DNA against radiations induced damage is depicted hypothetically in Figure 7. Natural polyphenolics already were shown to modulate gene expression, signal transduction cascades and DNA repair pathways [42–44]. Recently, REC-2006 was reported to contain several biologically active flavonoides, polyphenols and podophyllotoxin glycoside, and the like that might contribute toward enhancing DNA repair. These findings undoubtedly warrant further studies to unravel the effect of *P. hexandrum* on various DNA repair genes.

## 5. Conclusion

The results of our study imply that REC-2006 protect cellular DNA from radiation-induced damage by lowering the induction of the initial damage and by enhancing its repair *in vivo*. The antioxidant- and free-radical-scavenging properties of REC-2006, likely due to the presence of various bioactive compounds, may contribute toward its radioprotective effects.

## Figures and Tables

**Table 1:** Experimental Plan.

Group	Treatment	Time points after exposure (min)	Number of animals
1	Control	0	4
2	10 Gy	0	4
3	10 Gy	15	4
4	10 Gy	60	4
5	REC-2006 + 10 Gy	0	4
6	REC-2006 + 10 Gy	15	4
7	REC-2006 + 10 Gy	60	4
